# BH3-only proteins are dispensable for apoptosis induced by pharmacological inhibition of both MCL-1 and BCL-X_L_

**DOI:** 10.1038/s41418-018-0183-7

**Published:** 2018-09-05

**Authors:** Georgia Greaves, Mateus Milani, Michael Butterworth, Rachel J. Carter, Dominic P. Byrne, Patrick A. Eyers, Xu Luo, Gerald M. Cohen, Shankar Varadarajan

**Affiliations:** 1 Departments of Molecular and Clinical Cancer Medicine, Institute of Translational Medicine, University of Liverpool, Liverpool, Ashton Street, Liverpool, L69 3GE UK; 2 Department of Biochemistry, Institute of Integrative Biology, Crown Street, Liverpool, L69 7ZB UK; 3 Eppley Institute for Research in Cancer and Allied Diseases, Fred and Pamela Buffett Cancer Center, University of Nebraska Medical Center, Omaha, Nebraska 68198 USA; 4 Departments of Molecular and Clinical Pharmacology, Institute of Translational Medicine, University of Liverpool, Liverpool, Ashton Street, Liverpool, L69 3GE UK

**Keywords:** Cancer, Biochemistry

## Abstract

The impressive selectivity and efficacy of BH3 mimetics for treating cancer has largely been limited to BCL-2 dependent hematological malignancies. Most solid tumors depend on other anti-apoptotic proteins, including MCL-1, for survival. The recent description of S63845 as the first specific and potent MCL-1 inhibitor represents an important therapeutic advance, since MCL-1 is not targeted by the currently available BH3 mimetics, Navitoclax or Venetoclax, and is commonly associated with chemoresistance. In this study, we confirm a high binding affinity and selectivity of S63845 to induce apoptosis in MCL-1-dependent cancer cell lines. Furthermore, S63845 synergizes with other BH3 mimetics to induce apoptosis in cell lines derived from both hematological and solid tumors. Although the anti-apoptotic BCL-2 family members in these cell lines interact with a spectrum of pro-apoptotic BH3-only proteins to regulate apoptosis, these interactions alone do not explain the relative sensitivities of these cell lines to BH3 mimetic-induced apoptosis. These findings necessitated further investigation into the requirement of BH3-only proteins in BH3 mimetic-mediated apoptosis. Concurrent inhibition of BCL-X_L_ and MCL-1 by BH3 mimetics in colorectal HCT116 cells induced apoptosis in a BAX- but not BAK-dependent manner. Remarkably this apoptosis was independent of all known BH3-only proteins. Although BH3-only proteins were required for apoptosis induced as a result of BCL-X_L_ inhibition, this requirement was overcome when both BCL-X_L_ and MCL-1 were inhibited, implicating distinct mechanisms by which different anti-apoptotic BCL-2 family members may regulate apoptosis in cancer.

## Introduction

Abnormal cell survival through resistance to apoptosis is a cardinal feature of most malignancies and plays a key role in chemoresistance. [1, 2] As the major regulators of the intrinsic mitochondrial pathway of apoptosis, the pro-survival BCL-2 family proteins (BCL-2, BCL-X_L_, MCL-1, and BCL2A1) are attractive targets for novel cancer therapeutics. [3, 4] These proteins are proposed to function by binding and sequestering the pro-apoptotic BH3-only proteins, which in turn prevents the effector proteins, BAX and BAK, from forming pores in the mitochondrial membrane. The development of inhibitors of the pro-survival BCL-2 family proteins has proven particularly difficult because their inhibition requires disruption of these protein-protein interactions. However, after many years of research, small molecule inhibitors known as BH3 mimetics, which target the anti-apoptotic BCL-2 family have now been developed. The first clinically approved BH3 mimetic was ABT-263 (Navitoclax), an orally available form of ABT-737, which inhibits BCL-2, BCL-X_L_, and BCL-w [3, 5–7] and potently induces apoptosis in several solid and hematological cancers. This was followed by the introduction of a BCL-2-specific inhibitor, ABT-199 (Venetoclax), which has received approval for treating refractory chronic lymphocytic leukemia (CLL), where BCL-2 addiction is a key feature in the pathogenesis of the disease. [8–10] Recently, BH3 mimetics specifically targeting BCL-X_L_ (A-1331852) [11–15] and MCL-1 (A-1210477 and S63845) [1, 12, 16] have also been developed.

In this study, we demonstrate that S63845 is a highly potent and selective MCL-1 inhibitor, which can synergize with other BH3 mimetics, including ABT-199 and A-1331852, to induce apoptosis in a wide range of cell lines derived from different hematological malignancies and solid tumors. Furthermore, we demonstrate that BH3-only proteins are required for apoptosis induced following BCL-X_L_ inhibition, whereas dual inhibition of BCL-X_L_ and MCL-1 resulted in a BH3-only protein-independent cell death.

## Materials and methods

### Cell culture

Cell lines derived from mantle cell lymphoma (MAVER-1), diffuse large B cell lymphoma (U-2946), chronic myeloid leukemia (K562), acute myeloid leukemia (MOLM-13, OCI-AML-3, HL-60, MV-4-11, THP-1 and U-937), Jurkat-T-lymphocyte cell lines, wild-type and deficient in caspase-9 (from Prof. J. Borst, The Netherlands Cancer Institute-Antoni van Leeuwenhoek, Amsterdam), caspase-8 and FADD (from Prof. J. Blenis, Weill Cornell Medicine, USA), non-small cell lung carcinoma (H1299), prostate cancer (PC-3, from Prof. Y. Ke, University of Liverpool, UK) and triple negative breast cancer (MDA-MB-231 from P. Meier, Institute of Cancer Research, London, UK) were cultured in RPMI 1640 medium. H929, a multiple myeloma cell line, was cultured in the same medium supplemented with 0.02% 2-mercaptoethanol. The non-small cell lung carcinoma cell line A549 was cultured in DMEM/F12 supplemented with 1% non-essential amino acids. The pancreatic cancer cell line, SUIT-2 (from A. Mielgo, University of Liverpool, UK) was cultured in DMEM. Colon cancer cell lines HT-29 (from J. Parsons, University of Liverpool, UK) and HCT116 (wild-type, DKO and 8KO) [3, 17–22] as well as HCT116 cells deficient in BAX or BAK (from R. J. Youle, National Institute of Health, USA), were cultured in McCoys 5 A Modified media. All cell lines, unless otherwise specified, were either from DMSZ (Braunshweig, Germany) or ATCC (Middlesex, UK) and were validated using short tandem repeat profiling. Peripheral blood samples from CLL patients were obtained with patient consent and local ethics committee approval and cultured as described. [23] All media were supplemented with 10% fetal calf serum (Life Technologies Inc., Paisley, UK).

### Reagents

A-1331852 and A-1210477 from AbbVie Inc. (North Chicago, IL, USA), S63845 from ActiveBiochem (New Jersey, USA), and ABT-199 and Z-VAD.FMK from Selleck Chemicals Co. (Houston, TX, USA) were used. Antibodies against BCL-X_L_, BIM and BAD from Cell Signalling Technology (MA, USA), NOXA from Millipore (Watford, UK), BID and tubulin from Abcam (Cambridge, UK), MCL-1, BCL-2 and PUMA from Santa Cruz Biotechnology (Santa Cruz, CA, USA) and BAX and cytochrome *c* (BD BioSciences, California) were used for immunoblotting. Antibodies used for immunoprecipitation were MCL-1 (Y-37) and BCL-X_L_ from Abcam, BCL-2 from BD Biosciences and BAX (NT) from Merck-Millipore (Burlington, MA, USA). siRNAs specific to BCL-X_L_ (SI03025141), MCL-1 (SI02781205) and a non-targeting control (1027310) from Qiagen (Cambridge, UK) were incubated with Interferin siRNA transfection reagent (Polyplus transfection Inc., NY, USA) and added to cells at a concentration of 10 nM for 72 h.

### Differential scanning fluorimetry assay

Thermal shift assays were performed using a StepOnePlus Real-Time PCR machine (Life Technologies, Paisley, UK) with Spyro-Orange dye (Invitrogen, Paisley, UK) and purified recombinant BCL-2 (Abcam) and MCL-1 protein, as previously described. [3]

### Cytochrome *c* release, BAX translocation and apoptosis measurements

To quantitate cytochrome *c* release and BAX translocation, cells were grown on coverslips and treated with the specified inhibitors following a 0.5 h pre-treatment with the pan-caspase inhibitor Z-VAD.FMK (30 μM), and then fixed with 4% (v/v) paraformaldehyde and permeabilized with 0.5% (v/v) Triton X-100 in PBS, followed by incubation with primary and fluorophore-conjugated secondary antibodies prior to visualization under a fluorescent microscope. The extent of cytochrome *c* released from the mitochondria or BAX translocation to the mitochondria was quantified by counting at least 300 cells from three independent experiments. The extent of apoptosis in cells following different treatments was quantified using an Attune NxT flow cytometer (Thermo Fisher Scientific, Paisley, UK), as previously described. [24]

### Gel filtration, immunoprecipitation and Western blotting

Recombinant MCL-1 purification, size exclusion chromatography and immunoprecipitation experiments were carried out as previously described. [3, 25] Western blotting was carried out according to standard protocols. Briefly, 50 μg of total protein lysate was subjected to SDS-PAGE electrophoresis. Subsequently proteins were transferred to nitrocellulose membranes and protein bands visualized with ECL reagents (GE Healthcare).

### Clonogenic studies

Cells were seeded in 6 well plates at a density of 2000 cells/well and exposed to the specified inhibitors (100 nM) 24 h post-seeding. Cells were fixed on day 7 using methanol and acetic acid and then stained with crystal violet and colonies counted using the GelCount tumor colony counter (Oxford Optronix, Abbingdon, UK).

### Statistics

For time-course studies, a two-way ANOVA was performed using Dunnet’s multiple comparisons analysis and other studies were analyzed for statistical significance with one-way ANOVA using Sidak’s multiple comparisons analysis. The asterisks depicted correspond to the following *p* values: *p ≤ 0.05; **p ≤ 0.01; ***p ≤ 0.001.

## Results

### S63845 is a potent MCL-1 inhibitor and weak inducer of apoptosis in BCL-2- but not BCL-X_L_-dependent cells

**Fig. 1:**
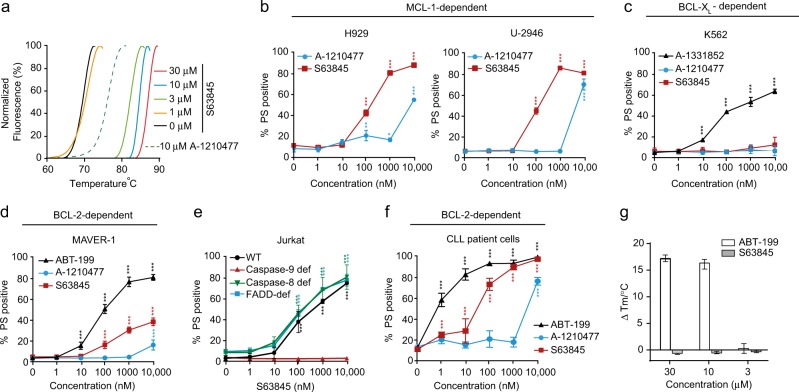
S63845 binds MCL-1 and induces apoptosis in several cancer cell lines. **a** S63845 exhibits a concentration-dependent shift in MCL-1 stabilization in a thermal stability assay. **b** S63845 and A-1210477 exhibit concentration-dependent apoptosis, as assessed by PS externalization using flow cytometry, in MCL-1-dependent cell lines, H929 and U-2946 after 4 h of exposure. **c** S63845 and A-1210477 fail to induce apoptosis in a BCL-X_L_-dependent cell line, K562, at 4 h, unlike A-1331852 (positive control). **d** MAVER-1, a BCL-2 dependent cell line, when exposed to the indicated BH3 mimetics for 4 h resulted in enhanced apoptosis. **e** Jurkat cells (wild type and deficient in the specified proteins), exposed to increasing concentrations of S63845 for 24 h exhibited caspase-9-dependent apoptosis. **f** Primary cells isolated from CLL patients, exposed to the indicated BH3 mimetics for 8 h, exhibited varying extents of apoptosis. **g** ABT-199 but not S63845 exhibits a concentration-dependent shift in BCL-2 stabilization in a thermal stability assay. Error bars = Mean ± SEM (standard error of the mean); *p ≤ 0.05; **p ≤ 0.01; ***p ≤ 0.001

Consistent with previous findings, [1] we demonstrated that S63845 is a potent MCL-1 inhibitor when compared with A-1210477, as assessed by a concentration-dependent thermal stabilization of MCL-1 in our in vitro thermal shift assay [3] (Fig. 1a). S63845 also induced rapid apoptosis in two MCL-1-dependent cell lines (H929 and U-2946), [3, 6] with an IC_50_~100 nM, demonstrating ~100-fold higher potency than A-1210477 (Fig. 1b). To assess the specificity of S63845 to induce apoptosis in a MCL-1 dependent manner, we exposed K562 cells (a BCL-X_L_-dependent cell line) and MAVER-1 cells (a BCL-2-dependent cell line) to increasing concentrations of S63845 (Fig. 1c, d). As expected, S63845 (as well as A-1210477) failed to induce apoptosis in K562 cells, whereas extensive apoptosis was observed following exposure to A-1331852 (Fig. 1c). However, S63845 (as well as A-1210477) induced a concentration dependent increase in apoptosis in MAVER-1 cells, albeit at higher concentrations than ABT-199 (Fig. 1d), suggesting that S63845 could either induce death in a non-specific manner or that it could also be a weak inhibitor of BCL-2. To test this, we exposed Jurkat cells (wild type as well as deficient in FADD, caspase-8 and caspase-9) to increasing concentrations of S63845. While S63845 induced similar levels of apoptosis in wild type as well as FADD/ caspase-8 deficient Jurkat cells, cells deficient in caspase-9 were completely resistant to S63845-mediated apoptosis (Fig. 1e), thus demonstrating that S63845 both specifically induced the intrinsic pathway of apoptosis and also did not induce non-specific cell death. To identify whether S63845, in addition to binding and inhibiting MCL-1, can also potentially bind and inhibit BCL-2, we exposed primary CLL cells that exclusively depend on BCL-2 for survival, [8] to increasing concentrations of S63845 and A-1210477. While ABT-199 (positive control) induced apoptosis in these cells at low nanomolar concentrations, both S63845 and A-1210477 also induced significant apoptosis at concentrations similar to their IC_50_ values in MCL-1 dependent cells (Fig. 1f). However, we did not detect any concentration-dependent thermal stabilization of BCL-2 following S63845 in an in vitro thermal shift assay, which was in marked contrast to the thermal stabilization of BCL-2 following ABT-199 in this assay (Fig. 1g). Taken together, our data demonstrates that under these experimental conditions, S63845 does not bind BCL-2 and induces apoptosis in a MCL-1-specific manner.

### S63845 is more potent than ABT-199 in inducing apoptosis in AML cell lines

**Fig. 2:**
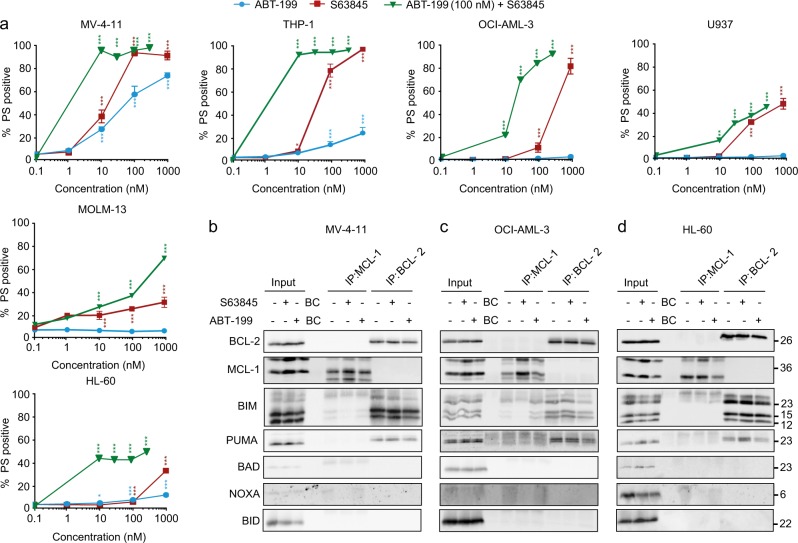
S63845 can synergize with ABT-199 to induce apoptosis in AML cell lines. **a** AML cell lines were exposed to a concentration range of S63845 with ABT-199 at a constant concentration of 100 nM for 24 h and assessed for PS externalization. Immunoprecipitation of MCL-1 and BCL-2 was carried out in **b** MV-4-11, **c** OCI-AML-3, and **d** HL-60 cells, following exposure to S63845 (30 nM for MV-4-11 and 100 nM for the other 2 cell lines) or ABT-199 (50 nM for MV-4-11 and 100 nM for the other 2 cell lines) for 6 h (MV-4-11) or 8 h (OCI-AML-3 and HL60). The eluted complexes were immunoblotted for the indicated proteins. The input represents the cell lysates and BC, beads control. Error bars = Mean ± SEM; *p ≤ 0.05; **p ≤ 0.01; ***p ≤ 0.001

Recent reports indicate that cells derived from acute myeloid leukemia (AML) patients can be effectively targeted with a BCL-2 and/or MCL-1 inhibitor, [11, 13–15] although other studies suggest that these cells depend primarily on BCL-2 for survival. [16] Indeed, MCL-1 inhibitors are currently entering clinical trials to treat AML and multiple myeloma patients (Clinical trials—NCT02979366; NCT02675452; NCT02992483). Therefore, we investigated whether S63845 alone or in combination with ABT-199 could induce apoptosis in a panel of AML cell lines. Of the six cell lines tested, MV-4-11 cells exhibited high sensitivity to both S63845 (IC_50_~20 nM) and ABT-199 (IC_50_ ~ 50-100 nM) individually. THP-1 was much more resistant to ABT-199 but sensitive to S63845 (IC_50_~50 nM). OCI-AML-3, U937 and MOLM-13 were completely resistant to ABT-199 but somewhat sensitive to S63845 at higher concentrations. In contrast, HL-60 cells were largely resistant to both BH3 mimetics individually (Fig. 2a). In the resistant cell lines, particularly U-937 and HL-60, BCL-2 and BCL-X_L_ appeared to perform redundant functions in cell survival, whereas MOLM-13 cells appeared to depend on BCL-X_L_ for survival, as exposure to A-1331852 alone resulted in significant apoptosis (Supplemental Fig. S1). Nevertheless, all AML cells examined were more sensitive to the combination of S63845 and ABT-199 (Fig. 2a).

### Interactions between the pro and anti-apoptotic BCL-2 family members do not reflect the relative sensitivities of AML cell lines to undergo BH3 mimetic-mediated apoptosis

BH3 mimetic-induced apoptosis is usually attributed to displacement of BH3-only proteins from their anti-apoptotic partners, leading to apoptosis. Since the different AML cell lines exhibited varied responses to BH3 mimetic-mediated apoptosis, we wished to assess if this could be attributed to altered binding and/or displacement of the different BH3-only proteins to their known survival counterparts. Therefore, we immunoprecipitated the anti-apoptotic proteins required for survival and probed for each of the BH3-only proteins (Fig. 2b–d). In MV-4-11 cells, which are sensitive to both ABT-199 and S63845, BIM and PUMA bound to BCL-2 and were slightly displaced following exposure to ABT-199 (Fig. 2b). In contrast, the traces of BIM and NOXA bound to MCL-1 in these cells were readily displaced after S63845 exposure, in-line with the extent of apoptosis observed with S63845 (IC_50_~20 nM). Neither BID nor BAD interacted with MCL-1 or BCL-2 in these cells (Fig. 2b). In OCI-AML-3, exposure to ABT-199 also resulted in a partial displacement of BIM and PUMA from BCL-2, despite the cells being largely resistant to ABT-199 (Fig. 2c). Similar to MV-4-11, BIM and NOXA bound to MCL-1 were readily displaced with S63845, and BID and BAD failed to interact with either MCL-1 or BCL-2 (Fig. 2c). In the resistant HL-60 cell line, BIM and PUMA bound to BCL-2 was not displaced following ABT-199, compatible with the cells being largely resistant to ABT-199 (Fig. 2d). BID and BAD were not bound to either MCL-1 or BCL-2. Moreover, of all the BH3-only members, only traces of NOXA appeared to interact with MCL-1 and was displaced following S63845 (Fig. 2d). Taken together, these results suggest that interactions between the pro- and anti-apoptotic BCL-2 family members may not necessarily reflect the relative sensitivities of different AML cell lines to undergo BH3 mimetic-mediated apoptosis.

### Interactions of BCL-X_L_ and MCL-1 with different BH3-only members differ in solid tumor cell lines

**Fig. 3:**
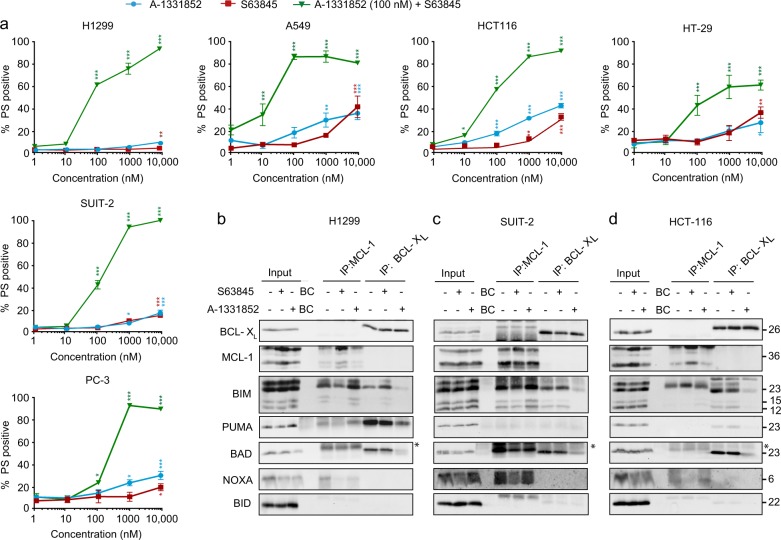
BH3-only proteins have different binding preferences in various solid tumor cell lines despite similar sensitivities to BH3 mimetics. **a** Solid tumor cell lines were treated with a concentration range of S63845 and a constant concentration of 100 nM of A-1331852 for 4 h and assessed for PS externalization. Immunoprecipitation of MCL-1 and BCL-X_L_ was carried out in H1299 **b**, SUIT-2 **c**, and HCT116 **d** cells exposed to S63845 (100 nM) or A-1331852 (100 nM) for 2 h, and the eluted complexes were immunoblotted for the indicated proteins. * in the blots denotes non-specific bands. Error bars = Mean ± SEM (standard error of the mean); *p ≤ 0.05; **p ≤ 0.01; ***p ≤ 0.001

Unlike several hematological malignancies, cancer cells derived from most solid tumors depend on both BCL-X_L_ and MCL-1 for survival. [3, 17, 19–22] Exposure of cells derived from a panel of solid tumors, including non-small cell lung carcinoma (H1299 and A549), pancreas (SUIT-2), colon (HCT116 and HT-29) and prostate cancer (PC-3) to a combination of S63845 or A-1331852 resulted in a marked induction of apoptosis, confirming that these cell lines depend on both BCL-X_L_ and MCL-1 for survival (Fig. 3a). To corroborate earlier findings in AML cell lines (Fig. 2) in non-hematological cells, we repeated immunoprecipitation studies and assessed interactions between the different BH3-only proteins with their key pro-survival counterparts, namely BCL-X_L_ and MCL-1. In H1299 cells, BIM and PUMA were preferentially bound to MCL-1 and BCL-X_L_, respectively, and were readily displaced following exposure to the relevant BH3 mimetics (Fig. 3b). This was particularly evident following exposure to S63845, in which the newly released BIM from the MCL-1 complex exhibited enhanced binding to BCL-X_L_. Similarly, exposure to A-1331852 displaced BIM from BCL-X_L_, which in turn facilitated its binding to MCL-1 (Fig. 3b). Other BH3-only proteins like NOXA and BAD exhibited selectivity in binding to MCL-1 and BCL-X_L_, respectively and were also displaced after exposure to the relevant BH3 mimetics. No detectable binding of BID to either BCL-X_L_ or MCL-1 was observed (Fig. 3b). In SUIT-2 cells, BIM was bound to both MCL-1 and BCL-X_L_ and was displaced to differing extents with the specific BH3 mimetics (Fig. 3c). In contrast to H1299, PUMA was not bound to either MCL-1 or BCL-X_L_ (Fig. 3c). However, there were some similarities, especially with NOXA being bound to MCL-1 and displaced with S63845, and BAD bound to BCL-X_L_, which could be disrupted following A-1331852 (Fig. 3c). Therefore, H1299 and SUIT-2 cells exhibit significant differences in their BCL-2 family interaction profile, despite similar expression levels of the different proteins and cell death responsiveness to the combination of A-1331852 and S63845. This was even more evident in HCT116 cells, in which most of the BH3-only proteins, with the exception of NOXA, interacted exclusively with BCL-X_L_, and could be readily displaced from their binding partners with A-1331852 (Fig. 3d). Collectively, these results suggest that the preferential sequestration of different BH3-only proteins to distinct anti-apoptotic counterparts does not solely dictate dependency of a cell line on an individual anti-apoptotic protein.

### BH3-only proteins are dispensable for BH3 mimetic-induced apoptosis in HCT116 cells

**Fig. 4:**
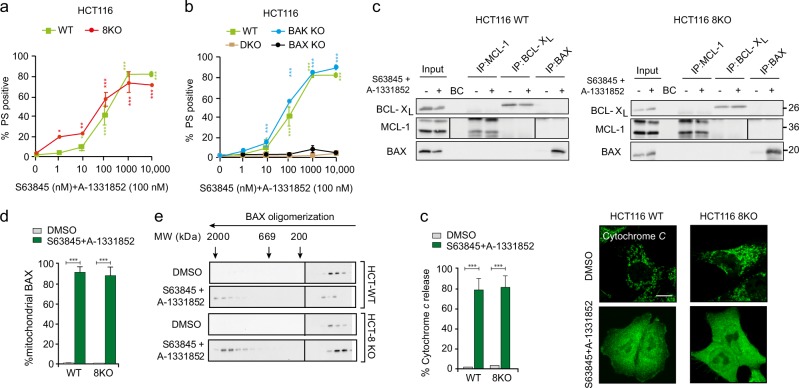
BH3 mimetics induce apoptosis even in the absence of BH3-only proteins in HCT116 cells. **a** HCT116 cells, wild-type (WT) and deficient in 8 different pro-apoptotic BH3-only proteins (8KO), were exposed to A-1331852 (100 nM) in combination with increasing concentrations of S63845 for 4 h and assessed for PS externalization. **b** Same as (**a**) but the experiments were carried out with HCT116 WT cells or cells deficient in BAX, BAK or both (DKO). **c** Immunoprecipitation of MCL-1, BCL-X_L_ and active-BAX in the indicated cells exposed to a combination of S63845 (100 nM) and A-1331852 (100 nM), following a 0.5 h pre-treatment with z-VAD.FMK (30 μM), were performed to assess BAX interaction. **d** The level of mitochondrial BAX in WT and 8KO cells following S63845 and A-1331852 treatment (both 100 nM) for 4 h was assessed by immunocytochemistry using an anti-BAX antibody. **e** Western blots of different molecular weight fractions from FPLC showing BAX oligomerization in HCT116 WT and 8KO cells upon exposure to S63845 (100 nM) and A-1331852 (100 nM) for 2 h. **f** Quantification and representative images of cytochrome *c* release in the indicated cell lines, from three independent experiments, following exposure to S63845 (100 nM) and A-1331852 (100 nM) for 4 h in the indicated cells. Error bars = Mean ± SEM (standard error of the mean); *p ≤ 0.05; **p ≤ 0.01; ***p ≤ 0.001

Since BH3-only proteins have recently been demonstrated to be dispensable for cell death, [18, 26] we evaluated whether selective inhibitors of BCL-X_L_ and MCL-1 could induce apoptosis in the absence of all known BH3-only proteins. Remarkably, in HCT116 8KO cells, which lack the 8 key BH3-only proteins, BIM, BID, PUMA, NOXA, HRK, BMF, BIK and BAD, [18] a combination of S63845 and A-1331852 induced apoptosis to the same extent as that of wild-type (WT) HCT116 cells (Fig. 4a). Furthermore, this combination of BH3 mimetics resulted in a BAX-dependent but BAK-independent apoptosis in these cells (Fig. 4b), emphasizing a crucial role for BAX in BH3 mimetic-mediated apoptosis. This raised the possibility of BAX directly interacting with BCL-X_L_ and/or MCL-1 to antagonize apoptosis. To test this hypothesis, we immunoprecipitated MCL-1 and BCL-X_L_ complexes from both HCT116 WT and 8KO cells but failed to detect BAX interaction with either anti-apoptotic protein (Fig. 4c). As a positive control, we immunoprecipitated active-BAX in these lysates, which pulled down BAX only following treatment with a combination of S63845 and A-1331852, indicative of BAX activation during apoptosis (Fig. 4c). These findings suggested that the BH3 mimetics could have displaced other protein(s) distinct from the 8 BH3-only proteins from BCL-X_L_ and/or MCL-1, which in turn activated BAX to facilitate mitochondrial outer membrane permeabilization (MOMP) and apoptosis. Apoptosis induction in the 8KO cells was accompanied by translocation of BAX from the cytosol to the mitochondrial membrane (Fig. 4d), BAX oligomerization into high molecular mass complexes as assessed by gel filtration (Fig. 4e), and MOMP, measured by the release of mitochondrial cytochrome *c* (Fig. 4f). Together, this negates a role for BH3-only proteins in several critical steps of the intrinsic apoptotic pathway induced by BH3 mimetics.

### Inhibition of BCL-X_L_ and MCL-1 results in BH3-independent apoptosis

**Fig. 5:**
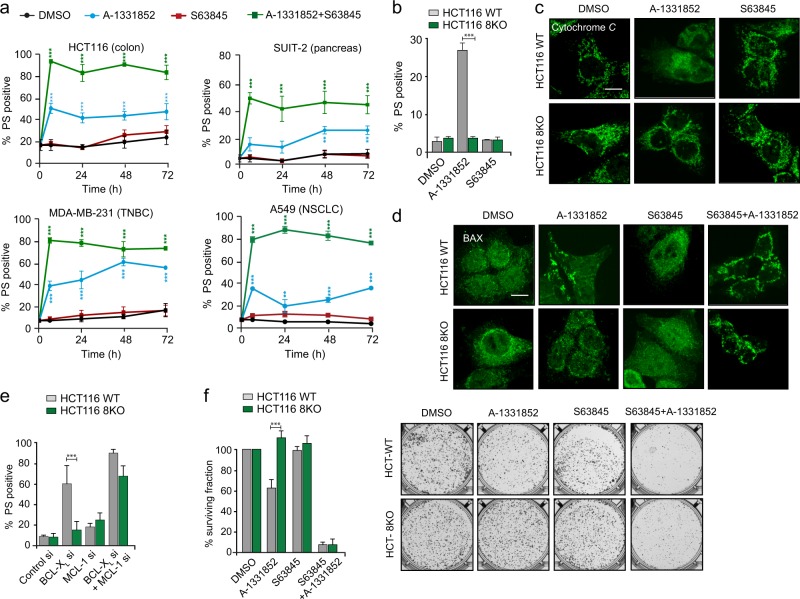
Apoptosis regulated by BCL-X_L_ requires BH3-only proteins. **a** Apoptosis was assessed by PS externalization in the indicated cell lines following exposure to A-1331852 (100 nM) and/or S63845 (100 nM) for the indicated times. HCT116 WT and 8KO cells were exposed to S63845 or A-1331852 (100 nM) for 24 h and assessed for **b** PS externalization, **c** cytochrome *c* release and **d** BAX translocation. For (**c**) and (**d**), cells were pre-treated with z-VAD.FMK (30 μM) for 0.5 h before exposure to BH3 mimetics. HCT116 WT and 8KO cells were **e** transfected with siRNA against BCL-X_L_ and/or MCL-1 for 72 h and assessed for PS externalization or **f** exposed to A-1331852 (100 nM) and/or S63845 (100 nM) and the number of colonies formed after 7 d was counted and images taken using GelCount. Representative images from the clonogenic assay are shown in the right panel. Error bars = Mean ± SEM (standard error of the mean); *p ≤ 0.05; **p ≤ 0.01; ***p ≤ 0.001

**Fig. 6:**
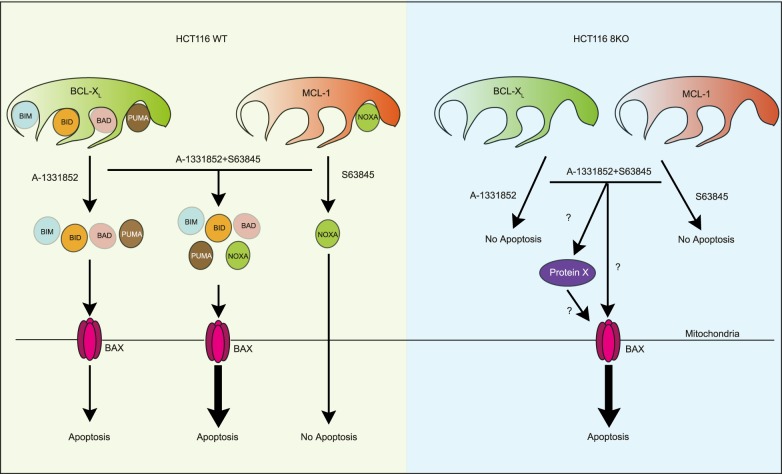
BCL-2 family members differ in their dependence on pro-apoptotic BH3-only members to exert their anti-apoptotic functions. In HCT116 cells, multiple BH3-only proteins, such as BIM, BID, BAD and PUMA are sequestered by BCL-X_L_, whereas NOXA is the only BH3-only protein bound to MCL-1. While the displacement of BH3-only proteins from BCL-X_L_ following A-1331852 is sufficient to induce apoptosis in these cells, displacement of NOXA from MCL-1 following S63845 failed to induce apoptosis. However, the combination of A-1331852 and S63845 released all the BH3-only proteins from the anti-apoptotic counterparts and resulted in pronounced apoptosis (bold arrow). In the absence of all eight BH3-only proteins, neither A-1331852 nor S63845 alone resulted in apoptosis. However, a combination of A-1331852 and S63845 still resulted in pronounced apoptosis, even in the absence of all BH3-only proteins. This could be due to the release of another pro-apoptotic BH3 domain-containing ‘protein x’ from MCL-1 and/or BCL-X_L_, which in turn could activate BAX in the 8KO cells. Alternatively, BH3 mimetics could indirectly result in the accumulation of BAX (either by inhibition of retrotranslocation or by passive diffusion, following the neutralization of the anti-apoptotic members) in the outer mitochondrial membrane, which in turn could result in BAX activation and apoptosis in the 8KO cells

Although many cell lines derived from solid tumors depend on both BCL-X_L_ and MCL-1 for survival, our studies revealed that inhibition of BCL-X_L_ following exposure to A-1331852 was sufficient to induce significant apoptosis in a panel of cell lines (Fig. 5a). To assess whether A-1331852-mediated apoptosis also occurred in the absence of all known BH3-only proteins, we employed HCT116 WT and 8KO cells to assess several hallmarks of apoptosis following BCL-X_L_ inhibition. Strikingly, exposure to A-1331852, but not S63845, resulted in enhanced apoptosis, as assessed by phosphatidylserine (PS) externalization, MOMP and mitochondrial translocation of BAX in HCT116 WT but not 8KO cells (Fig. 5b–d) suggesting that A-1331852-mediated apoptosis requires the presence of BH3-only proteins. Support for this suggestion was also provided by the enhanced apoptosis observed in WT, but not 8KO, cells following siRNA knockdown of BCL-X_L_ (Fig. 5e). However, this protective effect in 8KO cells was abolished when both BCL-X_L_ and MCL-1 were downregulated, suggesting a requirement of BH3-only proteins in BCL-X_L_ but not when both BCL-X_L_ and MCL-1 were downregulated. Similarly, exposure to A-1331852 significantly reduced the surviving fraction of WT, but not 8KO, cell populations (Fig. 5f). As expected, S63845 had no effect on clonogenic survival in either of the cell types, while a combination of the two BH3 mimetics obliterated clonogenicity in both cell types (Fig. 5f). Taken together, our data convincingly argue against the requirement of BH3-only proteins in apoptosis regulated by a combination of both BCL-X_L_ and MCL-1 (Fig. 6).

## Discussion

Prior to the discovery of S63845, putative MCL-1 inhibitors were poorly suited for clinical evaluation because they either lacked potency (high micromolar concentrations were required to inhibit MCL-1) or specificity resulting in undesirable toxicity. [3, 24, 27, 28] However, the development of S63845 potentially strengthens the use of a wide variety of BH3 mimetics in cancer therapy. In our hands, S63845 bound MCL-1 more extensively than A-1210477 and resulted in marked apoptosis in MCL-1-dependent cell lines (Fig. 1), and synergized with ABT-199 and A-1331852 to induce apoptosis in various cancer cell lines (Figs. 2 and 3), supporting the concept that a potent MCL-1 inhibitor, such as S63845, could be a valuable addition to the BH3 mimetic armamentarium.

Our finding that BH3-only proteins are dispensable for BH3 mimetic-mediated apoptosis in HCT116 cells is striking, particularly when the proposed mechanism of action of BH3 mimetics is taken into consideration (Fig. 4). Since apoptosis induction in these cells still required BAX and not BAK, [29] it is possible that BH3 mimetics could indirectly activate BAX by disrupting the interaction of BAX with BCL-X_L_ and/or MCL-1. However, we could not detect any BAX interaction with either of these anti-apoptotic proteins, despite efficient mitochondrial translocation and oligomerization of BAX, which resulted in MOMP and PS externalization (Fig. 4). Failure to detect BAX in complex with anti-apoptotic proteins could possibly be due to the dynamic nature of this interaction. Although BAX is generally considered to translocate from the cytosol to the outer mitochondrial membrane following apoptotic stimuli, several reports suggest a role for the anti-apoptotic BCL-2 family members in the constitutive retrotranslocation of BAX from the mitochondrial outer membrane to the cytosol even under non-apoptotic conditions. [30–35] Neutralization of the anti-apoptotic BCL-2 family members with BH3 mimetics could markedly reduce BAX retrotranslocation to the cytosol, [33, 36] thus retaining it in the outer mitochondrial membrane, which may facilitate direct activation of BAX (Fig. 6). [18] This could explain the apoptosis observed following BH3 mimetics in HCT116 cells, even in the absence of all known BH3-only proteins (Fig. 4).

Our data reveal that the requirement for BH3-only proteins in BCL-X_L_-regulated apoptosis can clearly be overcome when both MCL-1 and BCL-X_L_ are inhibited simultaneously (Figs. 4 and 5). Recent reports have demonstrated novel interacting partners of MCL-1, such as the BH3 domain-containing SUFU [37] and VLCAD, [38] which regulate distinct cellular functions. Therefore, other BH3-domain containing proteins distinct from the 8 key BH3-only members could interact with MCL-1 and/or BCL-X_L_ and play a role in these events (Fig. 6). In summary, our results demonstrate that BH3 mimetics induce apoptosis even in the absence of the eight best characterized BH3-only proteins, while also identifying differences in the regulation of cell survival by the different anti-apoptotic BCL-2 family members._._ This highlights the need to understand the fundamental mechanisms of apoptosis in order to improve therapeutic approaches using BH3 mimetics.

## Electronic supplementary material

Fig. S1

supplementary figure legends
