# Supplementary figures and images for: BH3-only proteins are dispensable for apoptosis induced by pharmacological inhibition of both MCL-1 and BCL-XL
Source: Cell Death Differ. 2018 Sep 5;26(6):1037–47. doi: 10.1038/s41418-018-0183-7 (PMC6748112; doi:10.1038/s41418-018-0183-7)

Figure S1

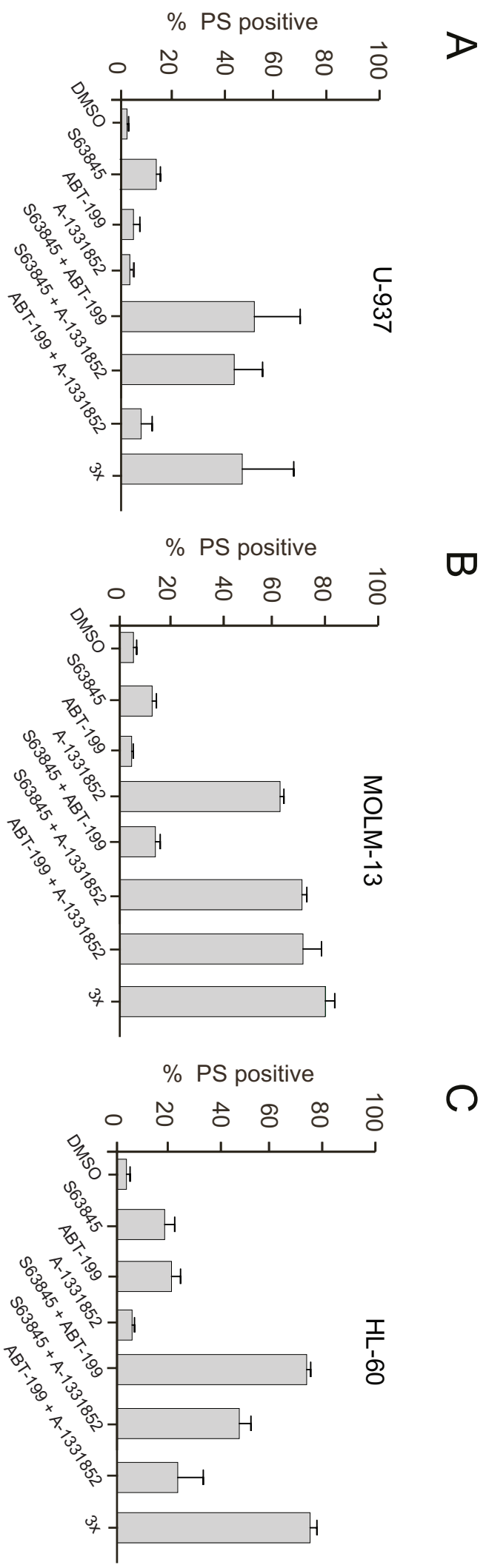

Supplement: Supplementary file 1 — Fig. S1 [file 41418_2018_183_MOESM1_ESM.pdf]
