# Supplementary material for: BH3-only proteins are dispensable for apoptosis induced by pharmacological inhibition of both MCL-1 and BCL-XL
Source: Cell Death Differ. 2018 Sep 5;26(6):1037–47. doi: 10.1038/s41418-018-0183-7 (PMC6748112; doi:10.1038/s41418-018-0183-7)
Supplement: Supplementary file 2 — supplementary figure legends [file 41418_2018_183_MOESM2_ESM.docx]

**Supplementary Figure Legend**

**Fig S1. AML cell lines resistant to BCL-2/MCL-1 inhibition exhibited varying sensitivity to A-1331852_._** (A-C) The indicated cell lines were exposed to the different BH3 mimetics (all at 100 nM) for 24 h and apoptosis assessed by PS externalization. In the figure, 3x represents a combination of all 3 BH3 mimetics. Error bars = Mean ± SEM.
